# Rationality, perception, and the all-seeing eye

**DOI:** 10.3758/s13423-016-1198-z

**Published:** 2016-12-07

**Authors:** Teppo Felin, Jan Koenderink, Joachim I. Krueger

**Affiliations:** 1 Saïd Business School, University of Oxford, Oxford, OX1 1HP UK; 2 Experimental Psychology, University of Leuven, Leuven, Belgium; 3 Experimental Psychology, Utrecht University, Utrecht, The Netherlands; 4 Cognitive, Linguistic, and Psychological Sciences, Brown University, Rhode Island, USA

**Keywords:** Rationality, Perception, Cognition, Social science

## Abstract

Seeing—perception and vision—is implicitly the fundamental building block of the literature on rationality and cognition. Herbert Simon and Daniel Kahneman’s arguments against the omniscience of economic agents—and the concept of bounded rationality—depend critically on a particular view of the nature of perception and vision. We propose that this framework of rationality merely replaces economic omniscience with perceptual omniscience. We show how the cognitive and social sciences feature a pervasive but problematic meta-assumption that is characterized by an “all-seeing eye.” We raise concerns about this assumption and discuss different ways in which the all-seeing eye manifests itself in existing research on (bounded) rationality. We first consider the centrality of vision and perception in Simon’s pioneering work. We then point to Kahneman’s work—particularly his article “Maps of Bounded Rationality”—to illustrate the pervasiveness of an all-seeing view of perception, as manifested in the extensive use of visual examples and illusions. Similar assumptions about perception can be found across a large literature in the cognitive sciences. The central problem is the present emphasis on inverse optics—the objective nature of objects and environments, e.g., size, contrast, and color. This framework ignores the nature of the organism and perceiver. We argue instead that reality is constructed and expressed, and we discuss the species-specificity of perception, as well as perception as a user interface. We draw on vision science as well as the arts to develop an alternative understanding of rationality in the cognitive and social sciences. We conclude with a discussion of the implications of our arguments for the rationality and decision-making literature in cognitive psychology and behavioral economics, along with suggesting some ways forward.

## Introduction

Our faculty of sight plays a central role in prominent theories of rationality—and assumptions about vision and perception lie at the very core of the cognitive, economic, and social sciences. For example, Herbert Simon’s breakthrough concept of bounded rationality challenged the idea of global rationality or omniscience in economics by focusing on “vision” and certain “psychological theories of perception and cognition” (Simon, 1956: 138). The ongoing behavioral and cognitive revolution in psychology and economics is also perception-centric and, as Kahneman says, “[relies] extensively on visual analogies” (2003a: 1450). More generally, it emphasizes visual illusions, visual tasks, and psychophysics (Tversky & Kahneman, 1986; cf. Kahneman, 1965). Assumptions about perception and vision are also at the very heart of a host of other theories of cognition across the social sciences, including Bayesian models of cognition and rationality (e.g., Chater et al., 2010: 813; Elqayam & Evans 2011; Oaksford & Chater, 2007; Tenenbaum & Griffiths, 2001; Vilares & Kording, 2011), research on decision making (e.g., Hilbert, 2012; Milkman et al., 2009; Payne et al., 1992; Shafir & LeBoeuf, 2002; Summerfield & Tsetsos, 2014), philosophy of mind (e.g., Block 2015; Burge, 2010), ideal versus naïve observer analysis (Geisler, 2008, 2011), rational expectations in economics (Kirman, 1992), theories of adaptive control and cognitive architecture (Anderson, 1996), universal models of cognition and optimal foraging (e.g., Fawcett et al., 2014; Hills et al., 2015; Pyke et al., 1977) as well as general models of “computational rationality” and intelligence (Gershman et al., 2015; Laird et al., 1987).

We argue that the literature on rationality features a unifying but problematic (and generally implicit) assumption about vision and perception that is best characterized by an “all-seeing eye” (cf. Koenderink, 2014; also see Hoffman, 2012; Hoffman & Prakash, 2014; Rogers, 2014). We focus particularly on how the all-seeing view of perception manifests itself in research on rationality, cognition, and decision-making. We point to the pioneering work of both Herbert Simon and Daniel Kahneman to illustrate our points (Kahneman, 2003a,b, 2011; Simon, 1956, 1980, 1990). Overall, the assumption of an all-seeing eye takes different forms across the social sciences. In some cases the all-seeing eye is assumed in the form of the rationality of some or all agents, or the system as a whole. In other cases the all-seeing eye is an emergent result of learning and visual, computational or information processing, or broader agent-environment interactions. In many cases the all-seeing eye is introduced in the form of the scientist who imputes illusion, bias, or other forms of error or veridicality to subjects—when they fall short of omniscience (Simon 1979; cf. Kahneman, 2003a). Each of these forms of all-seeing-ness, however, as we will illustrate, is problematic and is symptomatic of a representational, computational, and information processing-oriented conception of perception. In essence, much of the literature on rationality places an emphasis on psychophysics and inverse or ecological optics, ignoring the psychology and phenomenology of awareness (Koenderink, 2014). The emphasis is placed on the actual, physical nature of environments and objects within it (specifically, characteristics such as size, distance, color, etc)—rather than on the organism-specific, directed, and expressive nature of perception. We provide the outlines of a different approach to perception by drawing on alternative arguments about vision.

Our critique of extant work in the cognitive and economic sciences focuses explicitly on perception and vision, and thus is different from Gigerenzer’s (1991, 1996) approach, which emphasizes the “ecological” rationality of judgmental heuristics (Gigerenzer & Todd, 1999; Todd & Gigerenzer, 2012). The heuristics literature builds on a frequentist, Bayesian or “probabilistic view of perception” (Chater & Oaksford 2006; Kruglanski and Gigerenzer, 2011; Vilares & Kording, 2011), and more generally the “statistics of visual scenes” (Kersten et al., 2004; Knill & Richards, 1996; Yuille et al., 2004; cf. also Koenderink, 2016). The central argument in this literature is that perception, over time, is in fact veridical rather than biased: organisms perceive and interact with their environments and over time learn its true, objective nature. Though we link up with some of the ways in which this literature interprets (and indeed rightly questions) visual illusions, we also disagree with the way this work characterizes vision and perception, and point toward an alternative approach. We conclude with a discussion of how our arguments impact the rationality and decision-making literature in psychology and behavioral economics.

## Perception and cognition: From omniscience to bounded rationality

Any model of cognition, rationality, reasoning, or decision-making implicitly features an underlying theory of and assumptions about perception (Kahneman, 2003a; Simon, 1956). That is, any model of rationality makes assumptions about what options are seen or not, how (or whether) these options are represented and compared, and which ones are chosen and why. The very idea of rationality implies that someone—the agents themselves, the system as a whole or the scientist modeling the behavior—perceives and knows the optimal or best option and thus can define whether, and how, rationality is achieved. Rationality, then, is defined as correctly perceiving different options and choosing those that are objectively the best.

In emphasizing rationality, cognitive and social scientists are incorporating—most often implicitly—certain theories and assumptions about perception, about the abilities and ways in which organisms or agents perceive, see, and represent their environments, or compute and process information, compare options, behave, and make choices. Assumptions about perception and vision, as we will discuss, are at the very heart of these models and the focus of our paper.

Neoclassical economics has historically featured some of the most extreme assumptions about the nature of perception and rationality. This has taken the form of assuming some variant of a perfectly rational or omniscient actor and an associated “efficient market” (Fama, 1970; cf. Buchanan, 1959; Hayek, 1945).^1^ This work—in its most extreme form—assumes that agents have perfect information and thus there are no unique, agent-specific opportunities to be perceived or had: the environment is objectively captured and exhausted of any possibilities for creating value. Markets are said to be efficient as they, automatically and instantaneously, anticipate future contingencies and possibilities (Arrow & Debreu, 1954).

Much of this work assumes that there is, in effect, an “ideal observer” (cf. Geisler, 2011; Kersten et al., 2004)—either represented by the omniscience of all agents or the system as a whole—and thus an equilibrium (Arrow & Debreu, 1954). As noted by Buchanan, economists “have generally assumed omniscience in the observer, although the assumption is rarely made explicit” (1959: 126). The omniscient agent of economics has of course been criticized both from within and outside economics, as it does not allow for any subjectivity or individual level heterogeneity. For example, as Kirman argues, this approach “is fatally flawed because it attempts to impose order to the economy through the concept of an omniscient individual” (1992: 132). Thomas Sargent further argues that “The fact is that you simply cannot talk about differences within the typical rational expectations model. There is a communism of models. All agents inside the model, the econometrician, and God share the same model” (Evans & Honkapohja 2005: 566).^2^ Although the death of the omniscient agent of economics has been predicted for many years, it continues to influence large parts of the field.

It is precisely this literature in economics, which assumes different forms of global or perfect rationality, that led to the emergence of the behavioral and cognitive revolution in the social sciences, to challenge the idea of agent omniscience.^3^ Herbert Simon was the most influential early challenger of the traditional economic model of rationality. He sought to offer “an alternative to classical omniscient rationality” (1979: 357), and he anchored this alternative on the concept of “bounded rationality,” a concept specifically focused on the nature of vision and perception (Simon, 1956). Simon’s work was carried forward by Daniel Kahneman, who also sought to develop “a coherent alternative to the rational agent model” (2003a: 1449) by focusing on visual metaphors, illusions, and perception. We revisit both Simon and Kahneman’s work next.

To foreshadow our conclusion, we argue that both Simon and Kahneman, as well as later psychologists and behavioral economists, have unwittingly replaced the assumption of economic omniscience with perceptual omniscience, or an all-seeing view of perception. Neither Simon’s nor Kahneman’s model has overcome the paradigmatic assumption of omniscience, even though (or because) they have critiqued it. Instead, these models have merely introduced a different form of omniscience. We find it particularly important to revisit this work because it shows how the behavioral revolution was, and continues to be, deeply rooted in arguments about perception and vision. Though this work has sought to develop a psychologically more realistic and scientific approach to understanding rationality, we argue that this work can be challenged on both counts.

### Bounded rationality and perception

As noted above, Herbert Simon challenged the assumption of agent omniscience (particularly pervasive in economics) with the idea of bounded rationality. The specific goal of his research program was, to quote Simon again, “to replace the global rationality of economic man with a kind of rational behavior that is compatible with the *access to information* and the *computational capacities* that are *actually* possessed by organisms, including man, in the kind of environments in which such organisms exist” (1955: 99, emphasis added). Rather than presume the omniscience of organisms or agents, Simon hoped to interject psychological realism into the social sciences by modelling the “actual mechanisms involved in human and other organismic choice” (1956: 129). Bounded rationality became an important meta-concept and an influential alternative to models of the fully rational economic agent—a trans-disciplinary idea that has influenced a host of the social sciences, including psychology, political science, law, cognitive science, sociology, and economics (e.g., Camerer, 1998, 1999; Conlisk, 1996; Evans, 2002; Jolls et al., 1998; Jones, 1999; Korobkin, 2015; Luan et al., 2014; Payne et al., 1992; Puranam et al., 2015; Simon, 1978, 1980; Todd & Gigerenzer, 2003; Williamson, 1985). These notions of rationality continue to influence different disciplines in various ways, including recent work on universal models of reasoning, computation, and “search” (Gershman et al., 2015; Hills et al., 2015).

To unpack the specific problems associated with bounded rationality, as it relates to vision and perception, we revisit some of the original models and examples provided by Simon. We then discuss how these arguments have extended and evolved in the cognitive and social sciences more broadly (Kahneman, 2003a), including the domain of behavioral psychology and economics.

In most of his examples, Simon asks us to imagine an animal or organism searching for food in its environment (e.g., 1955, 1956, 1964, 1969; Newell & Simon, 1976; cf. Luan et al., 2014).^4^ This search happens on a predefined space (or what he also calls “surface”) where the organism can visually scan for food (choice options) and “locomote” and move toward and consume the best options (Simon, 1956). Initially the organism explores the space randomly. But it learns over time. Thus vision is seen as a tool for capturing information about and representing one’s environment.

What is central to the concept of bounded rationality, and most relevant to our arguments, is the specification of boundedness itself. Simon emphasizes the organism’s “perceptual apparatus” (1956: 130). The visual scanning and capturing of the environment for options is given primacy: “the organism’s *vision* permits it to see, at any moment, a *circular* portion of the surface about the point in which it is standing” (Simon, 1956: 130, *emphasis added*). Rather than omnisciently seeing (and considering) the full landscape of possibilities or environment (say, options for food)—as models of global rationality might specify things—Simon instead argues that perception (the relevant, more bounded consideration set of possibilities) is delimited by the organism’s “length and range of vision” (1956: 130-132). Similar arguments have recently been advanced in the cognitive sciences in universal models that emphasize perception and search (e.g., Fawcett et al., 2014; Gray, 2007; Luan et al., 2014; Todd et al., 2012).

One of Simon’s key contributions was to acknowledge that organisms (whether animals or humans) are not aware of, nor do they perceive or have time to compute, *all* alternatives in their environments (cf. Gibson 1979). Rather than globally seeing and optimizing, the organism instead “satisfices” based on the more delimited set of choices it perceives in its immediate, perceptual surroundings. Additional search, whether visually or through movement, is costly. Thus organisms search, scan, and perceive their environments locally and the tradeoffs between the costs of additional search and the payoff of choosing particular, immediate options drive behavior. In all, organisms only consider a small subset of possibilities in their environment—that which they perceive immediately around them—and then choose options that work best among the subset, rather than somehow optimizing based on all possible choices, which Simon argues would require god-like computational powers and omniscience.

These ideas certainly seem reasonable; but they are nonetheless rooted in a problematic conception of vision and perception. We foreshadow some central problems here, problems that we will more carefully address later in the paper when we discuss Kahneman’s (2003a,b) work and carefully revisit some of the common visual tasks and perceptual examples of bounded rationality and bias.

First, note that a central background assumption behind bounded rationality is that there is an all-seeing eye present which can determine whether an organism in fact behaved boundedly or rationally, or not. As Simon put it, “rationality is bounded when it falls short of omniscience” (1978: 356). For this shortfall in omniscience to be specified and captured, it requires an outside view, an all-seeing eye—in this case, specified by the scientist—that somehow perceives, specifies, computes, or (exhaustively) sees the other options in the first place, then identifies the best or rational one, which in turn allows one to point out the shortfall, boundedness or bias.

From the perspective of vision research, Simon’s “falling short of omniscience”-specification of bounded rationality can directly be linked to the “ideal observer theory” of perception (e.g., Geisler 1989, 2011; Kersten et al., 2004). Similar to the standard of omniscience, the “ideal observer is a hypothetical device that performs *optimally* in a perceptual task given the available information” (Geisler, 2011: 771, *emphasis added*). 5 Naïve (or bounded) subjects can be contrasted with a form of camera-like ideal observer who objectively records the environment. The comparison of objective environments with subjective assessments of these environments (or objects within it) has been utilized in the lab as well as in natural environments (Geisler, 2008; also see Foster, 2011; McKenzie, 2003). These approaches build on a veridical model of perception and objective reality, a sort of “Bayesian natural selection” (Geisler & Diehl, 2002) where “(perceptual) estimates that are nearer the truth have greater utility than those that are wide of the mark” (Geisler & Diehl, 2003). The environment is seen as objective, and subjects’ accurate or inaccurate responses are used as information about perception and judgment. This approach can be useful if we demand that subjects see something highly specific (whether they miss or accurately account for some stimulus specified by the scientist), though even the most basic of stimuli—as we will discuss—are hard to conclusively nail down in this fashion.

Extant work raises fundamental questions about whether perception indeed tracks truth (or “veridicality”) in the ways of an ideal observer (e.g., Hoffman et al., 2015). For example, evolutionary fitness maps more closely onto practical usefulness rather than any idea of truth or objectivity. Bayesian models of perception can be built on evolutionary usefulness rather than truth and accuracy (e.g., Hoffman & Singh, 2012; Koenderink, 2016). Supernormal stimuli highlight how illusory, seemingly objective, facts can be in the world (Tinbergen, 1951). We discuss these issues more fully later.

The problem is that the very specification of an objective landscape, space, or environment assumes that the scientist him or herself, in effect, is omniscient and has a god-like, true view of all (or at least a larger set of) options available to the organism under study—a type of third-person omniscience. The scientist sees all (or more) and can, *ex ante* and *post hoc*, specify what is the best course of action and whether the organism in fact perceived correctly, acted boundedly, or behaved rationally. But, in most cases, simply labelling something as biased or bounded does not amount to a theoretical explanation. Indeed, it serves as a temporary holding place that requires further investigation as to the reasons why something was perceived or judged in a certain way. Perhaps the organism simply did not have enough time to identify the optimal solution or the organism couldn’t see certain possibilities. The fact that perception and rationality consistently fall short of standards set forth by scientists raises questions not only about the standards themselves but also about *why* this is the case.

The second problem is that perception as seen by Simon is a camera-like activity where organisms capture veridical images of and possibilities in their environments and store or compare this information (cf. Simon, 1980). Granted, the camera used by organisms—perception and vision—is specified as bounded in that it captures only a small, delimited portion of the surrounding environment in which it is situated—that which can be immediately perceived (for example, “a circular portion” around an organism: Simon, 1956: 130)—rather than assuming omniscient awareness of the full environment. Whether only some or all of the environment is captured within the choice set of an organism, the approach assumes that perception generates objective representations or copies of the environment. Perception is equivalent to “veridical” or true representation, and only the bounds of what is perceived are narrowed, compared to the more omniscient models featured in economics and elsewhere. Simon et al.’s “CaMeRa” model of representation illustrates the point, specifically where “mental images resemble visual stimuli closely” (Tabachneck-Schijf et al., 1977: 309)—an assumption we will return to when discussing Kahneman’s more recent work. Perception as representation, and the efforts to map true environments to true conceptions of those environments, is the *sine qua non* of much of the cognitive sciences. Frequent appeals to learning, bias, boundedness, and limitations only make sense by arguing that there is a true, actual nature to environments (which can be learned over time).

The standard paradigm uses a world-to-mind, rather than a mind-to-world, model of perception that is, quite simply, not true to the nature of perception. Perception is not (just) representation (e.g., Purves, 2014) or world-to-mind mapping (Koenderink et al., 2014). The emphasis on representation places undue emphasis on the environment itself—and objects within it—rather than the organism-specific factors that in fact might originate and direct perception. Simon’s view of perception, then, falls squarely into the domain of psychophysics and inverse optics (cf. Marr, 1982): the attempts to map objective environments onto the mind. It implies a form of pure vision or veridical optics where the world can properly be captured and represented, if only there were enough eyes on it, or enough computational or perceptual power to do so (cf. Simon, 1955, 1956). Environmental percepts are treated as relatively deterministic and passive data and inputs to be represented in the mind.

The third and perhaps most central concern is the way that perception is implicitly seen as independent of the perceiver. Simon argues that the nature of the organism doesn’t meaningfully impact the argument, as highlighted by his interchangeable use of universal mechanisms applied to organisms in general, both animals and humans alike. For example, he argues that “human beings [or ants], viewed as a behaving system, are quite simple. The apparent complexity of his behavior over time is largely a reflection of the complexity of the environment in which he finds himself”’ (1969: 64-65). No attention is paid to the organism-specific factors associated with perception; the focus is on computation of perceived alternatives and the representation of an objective environment.^6^ Simon’s work was undoubtedly influenced in some form by behaviorism and its focus on the environment instead of the organism. He heralded the coming of a *universal* cognitive science (Simon, 1980, *Cognitive Science*), where a set of common concerns across “psychology, computer science, linguistics, economics, epistemology and social sciences generally”—focused on one idea: the organism as an “information processing system.” Perception, information gathering and processing provided the underlying, unifying model for this approach.^7^

The universality and generality of the arguments was also evident in Simon’s interest in linking human and artificial intelligence or rationality. In an article titled “the invariants of human behavior,” Simon argues that “since Homo Sapiens *shares some important psychological invariants* with certain nonbiological systems—the computers—I shall make frequent reference to them also” (1990: 3, *emphasis added*). He then goes on to delineate how human *and* computer cognition and rationality share similarities and are a function of such factors as sensory processing, memory, computational feasibility, bounded rationality, search, and pattern recognition. This approach represents a highly behavioral, externalist, and automaton-like conception of human perception and behavior (cf. Ariely, 2008; Bargh & Chartrand, 1997; Moors & De Houwer, 2006).

The concern with these arguments is that they do not recognize that perception is specific to an organism or a species—they instead assume a universality that has little empirical support. To suggest and assume that there is some kind of objective environment which the organism searches is not true to nature. Instead of generic or objective environments, organisms operate in their own “Umwelt” and surroundings (Uexkull 2010), where what they perceive is conditioned by the nature of what they are (Koenderink 2014). The work of Tinbergen and Lorenz in ethology makes valuable contributions by showing how organism-specific factors are central to perception and behavior. Yet, the standard paradigm bypasses the hard problem of perception—its specificity and comparative nature—by jumping directly to environmental analysis and by assuming that perception is universal and equivalent to inverse optics (the mapping of objective stimuli to the mind). Although we may seek to identify general factors related to objects, or environmental salience or objectivity across species, this simply is not possible as what is perceived is determined by the nature of the organism itself.

Simon’s notion of objective environments, which then can be compared to subjective representations of that environment, is also readily evident in a large range of theories across the domain of psychology and cognition. For example, in his influential *Architecture of Cognition,* Anderson (2013; also see Anderson & Lebieri, 2003, 2014) builds on precisely the same premise of universal cognition, in seeking to develop a “unitary theory of mind” focused on external representation and the mind as a “production system” (input-outputs and if-then statements driving organism interaction with the environment). This research builds on the longstanding “Newell’s dream” (Alan Newell, Herbert Simon’s frequent co-author) of building a computational and unified theory of cognition.

### Kahneman on perception

A timely example of how problematic models of perception and vision continue to plague the rationality and decision-making literature is provided by Kahneman’s Nobel Prize speech and subsequent *American Economic Review* publication (2003a) titled “Maps of Bounded Rationality.” A version of this article was also co-published in the *American Psychologist* (2003b). The article explicitly links the current conversations in cognitive psychology and behavioral economics with Simon’s work and our discussion in the previous section.

However, Kahneman’s work focuses even more directly on perception and vision. He argues that his approach is distinguished by the fact that “the behavior of agents is *not* guided by what they are able to compute”—à la Simon—“but by what they happen to *see* at a given moment” (Kahneman, 2003a: 1469, *emphasis added*). Sight thus takes center-stage as a metaphor for arguments about rationality. What is illustrative of Kahneman’s focus on perception and sight is that he “[relies] extensively on visual analogies” (2003a: 1450). The focal article in fact features many different visual tasks, pictures, and illusions, which are used as evidence and examples to make his points about the nature and limits of perception and rationality. We will revisit, and carefully reinterpret, some of these visual examples.

Kahneman’s emphasis on vision and perception is not all that surprising as his early work and scientific training—in the 1960s—was concerned with psychophysics, perception, and inverse optics: the study and measurement of physical and environmental stimuli. This early work focused on perception as a function of such factors as environmental exposure and contrast (Kahneman, 1965; Kahneman & Norman, 1964), visual masking (Kahneman, 1968), time intensity (Kahneman, 1966), and thresholds (Kahneman, 1967b). In other words, the study of perception is seen as the study of how (and whether) humans capture objects and environments based on the *actual* characteristics of objects and environments. These assumptions from Kahneman’s early work, and the broader domain of psychophysics, have carried over into the subsequent work on the nature of rationality. This view of perception is also center-stage in, for example, Bayesian models of rationality (e.g., Oaksford & Chater, 2010). The background assumption in all of this research is that “responding to [the actual attributes of reality] according to the frequency of occurrence of local patterns reveal[s] reality or bring[s] subjective values ‘closer’ to objective ones” (Purves et al., 2015: 4753).

In the target article Kahneman (2003a) conceptualizes individuals—similar to Simon—as “perceptual systems” that take in stimuli from the environment. As put by Kahneman, “the impressions that become accessible in any particular situation are mainly determined, of course, by the *actual properties* of the object of judgment” (2003a: 1453, *emphasis added*). This notion of perception explicitly accepts vision and perception as veridical or “true” representation (e.g., Marr, 1982; Palmer, 1999). Similar to Simon, the approach here is to build a world-to-mind mapping where “physical salience [of objects and environments] *determines* accessibility” (Kahneman, 2003a: 1453, *emphasis added*). Perception is the process of attending to, seeing, or recording—as suggested by Kahneman’s language of “impressions” and “accessibility” throughout the article— in camera-like fashion, physical stimuli in the environment based on the actual characteristics of objects and environments themselves.

The emphasis placed on the environment is evident in what Kahneman calls “natural assessments” (cf. Tversky & Kahneman, 1983). Natural assessments are environmental stimuli, characterized by the “actual,” “physical” features of objects that are recorded or “automatically perceived” or attended to by humans and organisms (Kahneman, 2003a: 1452). These physical features or stimuli include: “size, distance, and loudness, [and] the list includes more abstract properties such as similarity, causal propensity, surprisingness, affective valence, and mood” (Kahneman, 2003a: 1453). This work closely links with psychophysics: efforts to understand perception as a function of such factors as threshold stimuli or exposure (e.g., Kahneman, 1965).

Important to our arguments is that Kahneman equates perception—on a one-to-one basis—with rationality, intuition, and thinking itself, thus implying a specific environment-to-mind mapping of mind. This is evident in the claim that “rules that govern intuition are generally similar to the rules that govern perception,” or, more succinctly: “intuition resembles perception” (Kahneman, 2003a: 1450). Kahneman draws both analogical and direct links between perception and his conceptions of rationality, decision making, and behavior. Visual illusions, for example, are seen as instances and examples of the link between perception and the rationality. The discrepancy between what is seen (and reported) and what in fact is there, provides the basis for ascribing bias or irrationality to subjects. Visual illusions have thus become the example of choice for highlighting the bias and the limits of perception.

The assumed camera-like link between perception and cognition emerges across a wide range of literatures in the domain of rationality, reasoning, and cognition. For example, Chater et al. argue that the “problem of perception is that of inferring the structure of the world from sensory input” (2010: 813). Most Bayesian models of cognition, rationality, and decision making feature similar assumptions (cf. Jones and Love, 2011). The precise nature of these inferences, from a Bayesian perspective, is based on encounters with an objective environment, the nature of which can be learned with time and repeated exposure (cf. Duncan & Humphreys, 1989). The social sciences, then, are building on a broader psychological and scientific literature that treats “object perception as Bayesian inference” (Kersten et al., 2004; also see Chater et al., 2010). Bayesian perception compares observation and optimality (Ma, 2012; cf. Verghese, 2001), where the effort is to “accurately and efficiently” perceive in the form of “belief state representations” and to match these with some true state of the world (Lee, Ortega, & Stocker, 2014). Oaksford and Chater (2010) discuss this Bayesian “probabilistic turn in psychology” and the associated “probabilistic view of perception” in the social sciences, where repeated observations help agents learn about the true, objective nature of their environments. Bayesianism is now widely accepted, as Kahneman argues, “we know…that the human perceptual system is more reliably Bayesian” (2009: 523).^8^

### Revisiting and reinterpreting Kahneman’s examples

In the focal articles, Kahneman (2003a,b) provides five different visual illustrations and pictures to make his point about the nature and boundedness of perception and rationality. Scholars in the cognitive and social sciences have indeed heavily focused on visual tasks and illusions to illustrate the limitations, fallibility, and biases of human perception (e.g., Ariely, 2001; Gilovich & Griffin, 2010; Vilares & Kording, 2011). These visual examples are used to illustrate the (seeming) misperceptions associated with objectively judging such factors as size, color and contrast, context and comparison, and perspective. These examples are also used to point out perceptual salience and accessibility, the role of expectations and priming, and the more general problem of perceiving “veridically,” as an example of boundedness and bias (Kahneman, 2003a).

However, visual illusions are commonly misinterpreted (Rogers, 2014). First, they rarely provide a good example of bias in perception, but instead can be interpreted as illustrations of how the visual system works. Second, illusions and perceptual biases are simply an artefact of the problem of singularly and exhaustively representing objective reality in the first place. Thus we next point to some of Kahneman’s (2003a) examples and argue that these examples are wrongly interpreted, on both counts.

**Fig. 1:**
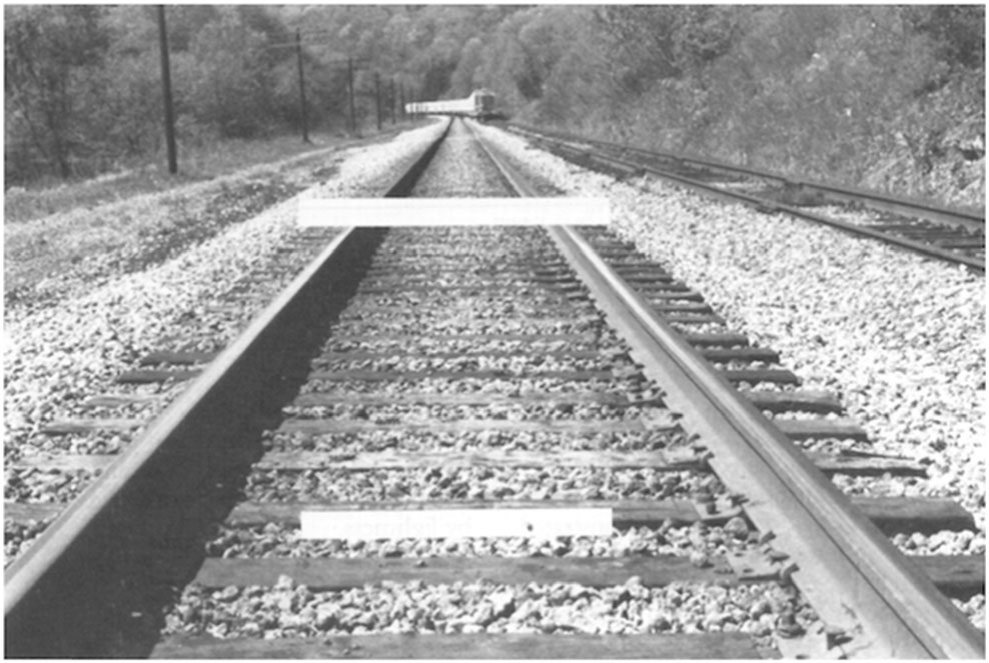
Ponzo illusion (from Gregory, 2005: 1243)

In one illustration, Kahneman (2003a: 1460) highlights the problem of accurately judging or comparing the size of objects by using a two-dimensional picture that seeks to represent a three-dimensional environment. Similar to the classic Ponzo illusion (see Fig. 1, copied from Gregory, 2005: 1243; cf. Ponzo, 1912), in the picture the focal objects (in the above case, the white lines) that are farther away (or higher, in the two-dimensional image) are seen as larger by human subjects, even though the objects are the same size on the two dimensional surface. Kahneman calls this “attribute substitution” and argues that the “illusion is caused by the differential accessibility of competing interpretations of the image” – and further that the “impression of three-dimensional size is the only impression of size that comes to mind for naïve observers—painters and experienced photographers are able to do better” (Kahneman, 2003a: 1461–1462). The perceptual naiveté of subjects, compared to experts, is indeed a popular theme in the rationality literature.

The problem is in how the visual task—that is purported to illustrate perceptual illusion and bias—is set up and how it is explained. The concern here is that the image features conflicting stimuli, namely, a conflict between the image and what it seeks to represent in the world. The reason that the top, white line in Fig. 1 (at first glance) appears to be longer is because the image features both two- and three-dimensional stimuli. Since the white line at the bottom (Fig. 1) is shorter than the railway ties it overlaps with—and the railway ties are presumed to be of equal length—it is natural to make the “mistake” of judging that the top line in fact is longer than the bottom line. The catch, or seeming illusion, is that the two white lines are of equal length in two-dimensional space. The issue is that the vertical lines disappearing into the horizon—the railroad tracks themselves—suggest a three-dimensional image, though the focal visual task relates to a two-dimensional comparison of the lengths of the two horizontal, white lines.

To illustrate the problem of labelling this an illusion, we might ask subjects whether the vertical lines (the rail road tracks) are merging and getting closer together (as they go into the horizon), or whether they remain equidistant. On a two-dimensional surface it would be correct to report that the lines are getting closer together and merging. This is how things appear in the image. But if the picture is interpreted as a representation of reality (of space, perspective, and horizon), then we might also correctly say that the lines are *not* getting closer together or merging. Furthermore, if the top, horizontal white line was in fact part of the three-dimensional scene that the picture represents, it would be correct to say that the top line indeed is longer. Experimental studies of visual space, using Blumenfeld alley experiments, provide strong evidence for the point that there is nothing straightforward about representing space on a two-dimensional surface or plane (e.g., Erkelens, 2015).

Furthermore, consider what would happen if subjects were asked to engage in the same task in a natural environment—rather than looking at a picture—standing in front of railroad tracks that go off into the horizon. What visual illusions could be pointed to in this setting? The subjects might, for example, report that the tracks themselves appear to remain equidistant and that the railroad ties appear to remain the same size. If the subjects slowly lifted up a 1-meter long stick, horizontally in front of them, at some point the stick would indeed be of seemingly equal (two-dimensional) length to one of horizontal railroad ties that are visible up further in the horizon.

We might briefly note that another interpretation of these types of perspective-based illusions is that they not only play with two and three dimensions, but that they also capture motion (e.g., Changizi et al., 2008). That is, human perception is conjectural and forward-looking, for example anticipating oncoming stimuli when in motion. Thus the converging or ancillary lines in the background of an image—commonly used in visual illusions (e.g., Ponzo, Hering, Orbison, & Müller-Lyer illusions)—can be interpreted as suggesting motion and thus appropriately “perceiving the present” and anticipating the relative size of objects.

Visual illusions are only *artificially* induced by taking advantage of the problem of representing a three-dimensional world in two dimensions. The discrepancies between two and three dimensions—the so-called data or evidence of visual illusions and bias—are not errors but simply (a) examples of how the visual system in fact works and (b) artefacts of the problem that two-dimensional representation is *never* true to any three-dimensional reality (we will touch on both issues below). The use of perspective-based visual illusions as evidence for fallibility, misperception, or bias is only a convenient tool to point out bias. But any bias is only the result of having subjects artificially toggle between representation and reality (or, more accurately, one form or expression of reality). To say that scientists have accurately captured some sort of bias is simply not true (Rogers, 2014). Visual illusions based on perspective are inappropriately exploiting and interpreting a more general problem, which is that two-dimensional images cannot fully represent three-dimensional reality. Moreover, as we will discuss, the very notion of appealing to some kind of singular verifiable reality as a benchmark for arbitrating between what is illusion or bias, versus what is not, is fraught with problems from the perspective of vision science (Koenderink, 2015; Rogers, 2014; see also Frith, 2007).

We might note that some scholars in the area of cognition and decision-making have recently noted that visual illusions are incorrectly used to argue that perception and cognition are biased. For example, Rieskamp et al. write: “Just as vision researchers construct situations in which the functioning of the visual system leads to incorrect inferences about the world (e.g., about line lengths in the Muller-Lyer illusion), researchers in the heuristics-and-biases program select problems in which reasoning by cognitive heuristics leads to violations of probability theory” (Rieskamp, Hertwig, & Todd, 2015: 222).

We agree with this assessment, but our point of departure is more fundamental and pertains to the nature of perception itself. Specifically, the extant critiques of bias (and associated interpretations of visual illusions) propose that humans eventually learn the true nature of the environment, and thus focus on alternatives such as a Bayesian probabilistic view of perception. But the problem is that “probability theory is firmly rooted in the belief in [an] all seeing eye” (Koenderink, 2016: 252). In other words, the idea of Bayesian “ecological rationality” (Goldstein & Gigerenzer, 2002; Todd & Gigerenzer, 2012) builds on a model of ecological optics (cf. Gibson, 1977)—where perception is also seen in camera-like fashion: humans learn the true nature of environments over time. The notion of ecological rationality and optics implies that illusions are mere temporary discrepancies between representations and the real world. We propose a fundamentally different view, one that suggests it is not as easy (if not impossible) to disentangle illusion, perception, and reality. Thus, while we agree with the critique, our point of departure anchors on a very different view of perception, which we will outline in the next section.

**Fig. 2:**
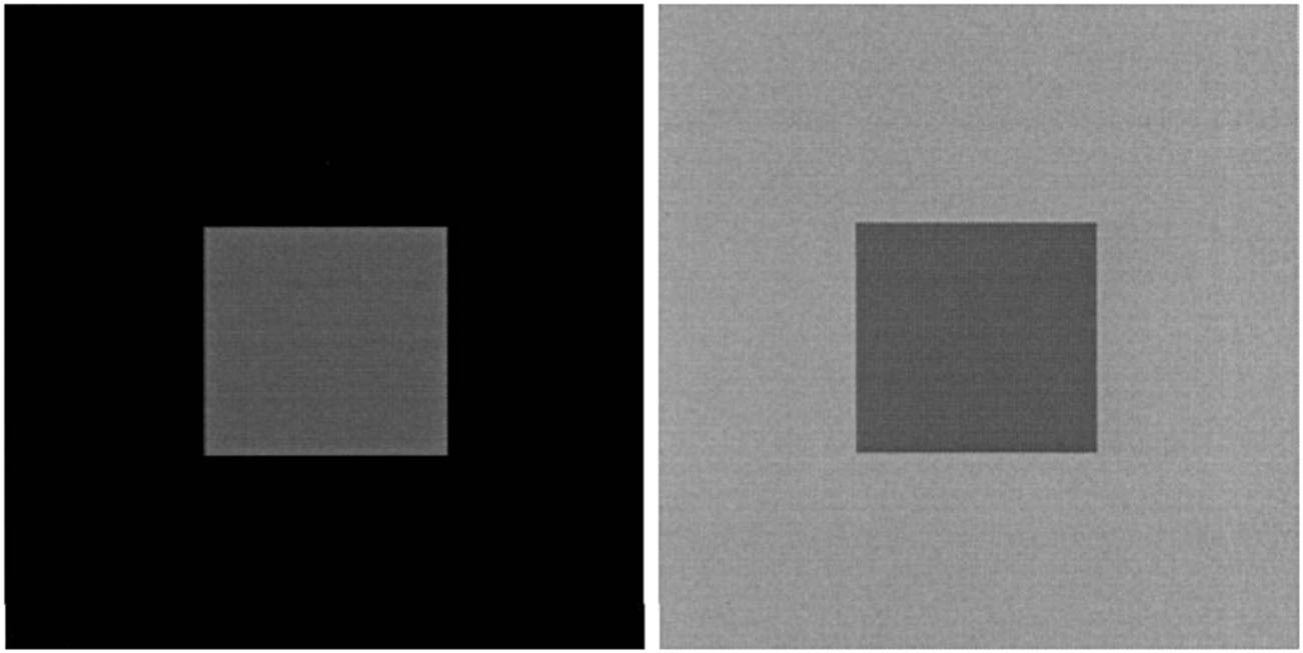
Perception of brightness (from Kahneman, 2003: 1455)

To illustrate further concerns with how perception is treated in this literature, we focus on another visual example provided by Kahneman (see Fig. 2 – from Kahneman, 2003a: 1455). This example is used by Kahneman to show the “reference-dependence of vision and perception” (2003a: 1455). He specifically points to reference-dependence by discussing how the perception of brightness or luminance can be manipulated by varying the surrounding context within which the focal image is embedded (see Fig. 2 – from Kahneman, 2003a: 1455). In other words, it would appear that the inset squares in Fig. 2 differ in brightness, due to the varied luminance of the surrounding context. But the two inset squares in fact have the same luminance. Kahneman thus argues that the “brightness of an area is not a single-parameter function of the light energy that reaches the eye from that area” (2003a: 1455). Stopping short of calling this an illusion, the implication is that the reference-dependence of vision says something about our inability to judge things objectively and veridically, even though actual luminance in fact can be objectively measured.^9^ A wide variety of brightness and color-related illusions have of course been extensively studied by others as well (Adelson, 1993, 2000; Gilchrist 2007).

The concern with this example is that the use of color or luminance tasks artificially exploits the fact that no objective measurement of color or luminance is even possible (Koenderink, 2010).^10^ Using shadows or changing the surrounding context or luminance of a focal image, a common approach to pointing out illusions, is not evidence that perception itself is biased or illusory. Kahneman is correct when he says that color or luminance is “reference-dependent.” But the underlying assumption remains that there also is a true, objective way to measure luminance itself—by the scientist—and to highlight how human judgment deviates from this objective measurement. Unfortunately no such measurement is possible for color (Koenderink, 2010; cf. Maund, 2006).

As discussed by Purves et al., any “discrepancies between lightness and luminance…are not illusions” (2015: 4753). We may infer that the “true” state of luminance is not observed by a subject (Adelson, 1993), but any observation, measurement, or perception is *always* conflated with a number of factors that cannot fully be separated (Koenderink, 2010). We may only care about the focal retinal stimulus itself, but perception and observation is also a function of illumination, reflectance, and transmittance (Purves et al. 2015). These factors are all inextricably conflated in a way that makes it impossible to extract true measurement (Koenderink, 2010). Similar to perspective-based visual illusions (where the illusion is artificially created by exploiting the gap between two-dimensional representation and three-dimensional reality), with luminance-based tasks scientists are only tricking themselves in pointing out observational discrepancies between perception and reality, rather than meaningfully pointing out bias. Color and luminance are always confounded by context (which includes a host of factors), and no objective measurement is possible (cf. Gilchrist et al., 1999; Gilchrist, 2006). Kahneman would seem to agree with this when he notes the context-dependence of perceptions. But his underlying “veridical” approach to perception and vision is in direct conflict with this argument (Kahneman, 2003a: 1460).^11^

Most importantly, the nature of the perceiver matters. As discussed by Rogers, “there can be no such thing as ‘color information’ that is independent of the perceptual system which is extracting that information” (2014: 843). The way color or luminance is perceived depends on who and what, in what context, is doing the perceiving. The human visual system is highly specific—that is, it sees or registers a select portion of the light spectrum, responding to wavelengths between 390 nm and 700 nm. We wouldn’t point to illusion or bias if someone were not able to see spectra outside this range, for example, ultraviolet light – which can be measured. As discovered by Newton, we see some aspects of light or color but not others. Chromatic aberrations highlight how white light includes a spectrum of colors. Indeed, the very idea of “light” could be cast as an illusion, as alternative realities (e.g., the color spectrum) can be measured and proven. Of course, any discussion of color needs to wrestle with and separate colorimetry and the phenomenology of light and color (Koenderink, 2010).

Note also that the way that any particular, seemingly objective color is represented or subjectively sensed varies across species. A bat sees the world very differently than humans do (cf. Nagel, 1974). Luminance or color has no “true” or objective nature (Koenderink, 2010). It is mental paint. Different species not only see the same colors differently, or don’t see them at all, but they have different *interpretations* of the very same inputs, stimuli, and data. Furthermore, the human’s built-in mechanism for maintaining color constancy should not be regarded as an illusion (cf. Foster, 2011), though it is often used as such (cf. Albertazzi, 2013). For example, in the real world we assume color constancy in the presence of shadows, even though this information can wrongly be used as evidence for illusion or bias when judging luminance or color in pictures (Adelson, 2000; cf. Gilchrist, 2006; Purves, 2014; Rogers, 2014).

In all, although we can measure (and thus “objectively” show the existence of) a large range of possible frequencies across the electromagnetic spectrum, with various instruments, nonetheless the human visual system allows only certain types of input. This is true not only for luminance, but also for many other visual and perceptual factors. This very argument casts doubt on any one way of measuring perception and reality in the first place—an argument we will turn to next.

## An alternative approach to perception

Throughout this manuscript—in criticizing extant conceptions of perception and rationality—we have broadly alluded to some ways forward. We now outline an alternative approach to perception and subsequently discuss its implications for the study of human judgment and decision-making as well as models of rationality.

The core of our argument is that perception and vision is species-specific, directed, and expressive instead of singular, linear, representative, and objective. We are not the first to question the assumption of an all-seeing view of perception; yet much extant work across the cognitive sciences continues to rely on this assumption. Perception necessarily originates from a perspective, or point of view.

### Organism-specific perception

The focus on the limits, errors, and boundedness or bias in perception misses a fundamental point about perception, namely that perception is organism- and species-specific. In an effort to develop *general* models of cognition and rationality (across different organisms, and even to account for artificial intelligence: Simon, 1980, 1990), scholars have lost sight of central insights from domains such as ethology. Ethology is the branch of biology that focuses on species-specificity, the comparative *nature* of organisms. Instead of attempting to generate models “claimed to be general” (Tinbergen, 1963: 111), ethology is concerned with the *comparative* and *unique* nature of organisms, in terms of vision, perception, the senses, rationality, behavior, and any number of other domains (Lorenz, 1955; for a historical review see Burkhardt, 2005).

One of the pioneers of the ethological approach to perception was the biologist Jakob von Uexküll (1921; 2011; cf. Riedl, 1984). Von Uexküll argued that each organism has its own, unique “*Umwelt,*” by which he meant the context of existence. He noted that “every animal is surrounded with different things, the dog is surrounded by dog things and the dragonfly is surrounded by dragonfly things” (2010: 117). These *Umwelten* or surroundings are not objective, but they comprise what the organism attends to, sees, and ignores. Hence, *Umwelten* vary across species and even across individual organisms within a species.

Any object in an environment—say, a tree—is and *means* very different things, depending on the observer or species in question. A tree is a place of shelter for one species, a nesting location for another, an object of beauty, an obstacle, shade, a source of food, or a lookout point. The list of possible “affordances” for any object is long (Uexküll 2010; cf. Gibson, 1979). Importantly, different aspects of “tree” are visible to different species. Awareness is not conditioned by what is there, but by the nature of the observer. Some focus on or simply see a particular color and others focus on, say, size. To give an example from another context, stickleback fish are attracted by and attuned to the color red, at the expense of seeing other more “real” features of potential mates (Tinbergen, 1951). What is perceptually “selected” or attended to or seen—which units, portions, and boundaries are relevant to the organism—varies significantly. Perception therefore depends more on the nature of the organism than on the nature of the environment. We cannot point to a single, objective characteristic of an object (whether color or size, as is done in psychophysics) or environment to capture some form of true perception. Although there are overlaps in both affordances and in what is perceived (what might be called “public objects”; Hoffman, 2013), species see things in radically different ways.

Perception requires a deeper “grammar,” an understanding of the nature of the perceiving organism itself. Similar to language learning (Chomsky, 1957), we can focus on and measure environmental inputs—exposure, repetition, and stimuli to explain, say, language, as the behaviorists did—or we can focus on the underlying, latent, developing, and species-specific capacity for language *despite* impoverished inputs or stimuli. No amount of exposure to linguistic or perceptual stimuli—no matter how frequent or how intense—will create the capacity to speak or perceive something if the underlying capacity or nature to receive those stimuli does not exist in the first place. To provide a stylized example: if a child carried around a hypothetical pet bee throughout its childhood, both child and bee would be exposed to the same environments, percepts, and stimuli. Yet, the child would not develop the navigational abilities of the bee and the bee would not develop the language or perceptual capacities of the child (Chomsky, 2002). Each would have very different—neither right nor wrong, but different—perceptions of their environments.

Perception requires an ability and readiness to respond to relevant stimuli (Mackay, 1969). The problem of perception has, instead, in the rationality literature, been framed as one of needing to deal with—or somehow properly compute, capture, or see—the overwhelming inputs or correct, external stimuli and to represent the world in accurate ways (cf. Kahneman, 2003a,b). But a more fundamental issue is the directedness of perception due to *a priori* factors associated with the organism itself. In psychology there is indeed a parallel program of research which focuses on perception and the *a priori* or “core” knowledge of humans, in reaction to extant empiricist, “periphery-inward”-type, input-output models of perception and behavior (e.g., Spelke, et al., 1992).

Stepping back, our intent is to focus on a different way of conceiving the nature of organisms, with particular attention to perception and vision. As noted by Simon, the appropriate specification of the underlying nature of organisms is indeed a fundamental starting point for any scientific analysis: “Nothing is more fundamental in setting our research agenda and informing our research methods than our view of the *nature* of the human beings whose behavior we are studying” (1985: 303). This underlying nature, for Simon and in subsequent work by Kahneman and others, focuses on perceptual boundedness, inputs and outputs, and computational limitation—generating models of rationality that can be verified against objective realities. We agree with Simon that the underlying specification of human nature matters. But we argue for a radically different, organism-specific understanding of nature, perception, and rationality.

### Perception as a user interface

A powerful way of thinking about perception (and objects or environments) is as a species-specific user interface (Hoffman, 2009; Hoffman et al., 2015; Koenderink, 2011, 2015). What organisms, humans included, perceive is not the actual nature of things. As noted by Frith, “we do not have direct access to the physical world. It may feel as if we have direct access, but this is an illusion created by our brain” (2007: 40; cf. Kandel, 2013). Perception and vision thus is, in effect, a species-specific interface that presents salient objects and features.^12^ What is visible on the interface—the way that objects or “icons” are, or how they are perceived—can be thought about as species-specific mental paint. Just as a computer’s interface doesn’t match any actual reality (and icons could vary wildly in, for example, color), and in fact is an illusion, so perception is some part illusion (or hallucination)—albeit a very useful illusion. The perceptual interface hides much of reality behind a set of things that are salient to a species. The fact that many aspects of reality are hidden is useful rather than a computational problem or lack of objectivity on the part of the organism or observer. The perception of particular objects also reflects the specific nature and capability of any organism. The lack of a capacity to see something as “x” and not as “y”—just as any species-specific capacity: bird-like flight, bat-like echolocation, or bee-like navigation—is not somehow problematic, or data to be utilized for highlighting bias or boundedness, but simply inherent to the nature of the organism itself.

The notion of perception as a user interface reinforces our claim that there is no possible way to point to or verify any *one* objective reality against which we might test susceptibility to illusion or bias. Any discussion of color or luminance illustrates this. For all practical purposes we can treat color as real in our day-to-day interactions and behavior, without getting into details about color spectra, the phenomenology of color, the nature of light or electromagnetic waves, and radiation (cf. Wilczek, 2016). In other words, our perceptual interface is useful and serves us quite well, without having to get into the actual physical or objective nature of things (as is done in the rationality literature). The problem is that even the most real, tangible, and physical of objects—say, a table—is not verifiable in a scientific sense (though pragmatically we of course see it), despite the physicalism and materialism emphasized by many in science. Just as a laptop provides a useful, perceptual interface that hides other realities (which in turn hide yet other realities), so a table or any other physical thing can be seen as a species-specific icon. As discussed by Eddington (1927: 11–16), a physical object such as a table is not just what we see (and any physical features we might ascribe to it or measure: color, size, weight), but it is also—counter to what is visible to us—largely made up of “empty space.” Even the most basic or essential of actual, physical elements, an atom, in fact “has no physical properties at all” (see Heisenberg, 1933; also Bell, 1990; Wilczek, 2016). In modern physics—compared to classical physics—there are neither any meaningfully physical properties (e.g., Mermin, 1998; Mohrhoff, 2000) nor any form of objective observer-independence (e.g., Bub, 1999; Maudlin, 2011; Wilczek, 2016).

A problem is then introduced by the demands that existing work on rationality places on the “physical” and “actual properties of the object of judgment” (Kahneman, 2003; cf. Chater et al., 2010; Kersten et al., 2004). These actual properties are impossible to pin down, due to their multidimensionality. We might say that focusing on the actual, objective reality simply represents a pragmatic and empirical stance: objectivity only applies to what humans can actually touch and see (or verify)—thus circumventing any discussions that might get into metaphysics or the nature of reality. But as our discussion of various visual tasks and examples illustrates (e.g., luminance and visual illusions), it is impossible to point to any one true way that things really are.^13^ Objects can be seen, described and represented in a *large variety of ways*—as we’ll further emphasize next. We may be able to momentarily trap subjects into seeming illusions, into not seeing things in one specific and rational way that we might demand of them. But these illusions are only an artefact of demanding that perception conforms to one point of view, even though other views are possible, depending on the perspective.

Rather than anchor on any form of computation or environmental and camera-like representation, our focus is not just on the species-specificity and user interface nature of perception, but also on the directedness of perception. This idea of the directedness of perception might informally be captured by Popper’s (1972) contrast between bucket theories of mind versus searchlight theories of mind. Bucket theories represent a stimulus and input-/output-oriented model of mind where environmental information and perceptions are passively and automatically—without meaning (cf. Koenderink et al., 2015; Pinna, 2010; Powell, 2001)—poured in as a function of exposure, the actual nature of stimuli, and experience. The searchlight model of mind assumes that perception is driven by the set of guesses, questions, conjectures, hypotheses, and theories that the mind (or organism) brings to the world (cf. Brown, 2011; Koenderink, 2011). The notion of a searchlight theory of mind might be compared to the idea of “perception as hypotheses” (cf. Gregory 1980). From this perspective, perception is actively directed toward certain features and it is expressive. Perception is not a process of identifying or learning some set of capital-T truths about environments, and objects within it, but rather an emphasis is placed on the organism-specific factors that direct perception and attention.

In contrast, the “what you see is what you get”-approach to perception (Hoffman, 2012) treats vision “as an inverse inference problem” (Yuille & Kersten 2004), where the visual system seeks to “match the structure of the world” (Knill et al., 1996: 6). This approach treats perception as an effort to map “sensory input to environmental layout” (Chater et al. 2006: 287), or sees it as an effort to infer “the structure of the world from perceptual input” (Oaksford and Chater, 2007: 93). But the efforts to map the external world onto the mind cannot be retrofitted into the perspective that we are suggesting here. Some argue that the idea of perception as a user interface is simply a version of Bayesian perception (Feldman, 2015). This argument is that perception does not track capital-T truth or beliefs in the world, but that perception tracks usefulness and that by doing so leads to fitness and improved performance for organisms and species. But focusing on usefulness, instead of truth, is fundamentally in violation of the underlying assumptions and foundation of Bayesian approaches to perception and vision (Hoffman & Singh, 2012; Hoffman et al., 2015).

### Perception, perspective, and art

The problems and opportunities encountered by artists and scholars who study the psychology and perception of art provide a useful window into the nature of vision (cf. Arnheim, 1954; Clark, 2009; Gombrich, 1956; Grootenboer, 2005; Helmholtz 1887; Hyman, 2006; Ivins, 1938; Koenderink, 2014; Kulvicki, 2006, 2014; Panofsky, 1955, 1991). In this section we show how the arts teach us that *any* attempts at veridical representation and perception necessarily result in illusion (cf. Kandel, 2012). We concur with Arnheim who wrote that “perception turns out to be *not* a mechanical recording of the stimuli imposed by the physical world upon the receptor organs of man and animal, but the eminently active and creative grasping of reality” (1986: 5). No true representation—more specifically, no *single* objective representation—is possible as there are many possibilities for representing reality (Koenderink et al., 2015; Rauschenbach, 1985). Placing an emphasis on any one element when seeking to represent reality necessarily means that other parts are not represented. Any one representation is just that: one representation chosen amongst a very large set of possibilities. Reality can be expressed in many ways. Various potential representations and expressions are not necessarily mutually exclusive, but useful for particular purposes, making different features salient. Thus it is hard to distinguish whether one representation is better or more veridical than another. Instead we might look for veridicality on certain dimensions (for example, whether three dimensions are appropriately captured), or better yet, for usefulness for making certain features salient.

Perhaps the best way of illustrating the problem of perception and representation, as informed by the arts, is by focusing on “linear” perspective and the aforementioned problem of capturing three-dimensional reality on a two-dimensional surface (Kandel, 2012; Mausfeld, 2002). The delineation of a Euclidean space allows three dimensions to be represented on a two dimensional surface (cf. Koenderink, 2012). This is done by taking a fixed position, a point of view, and then identifying a vanishing point—a horizon where vertical, parallel lines meet—where distance is represented by size and convergence.

The problem is that the use of Euclidean space and vanishing points on a two-dimensional canvas necessarily produces an illusion, as the vertical lines do not in fact converge (e.g., the railroad tracks in Fig. 1). Incorporating distance and space into a representation is beyond the capacity of the medium (a two-dimensional surface), necessitating illusion and the omission of other aspects of reality. As vividly articulated by the Russian mathematician Pavel Florensky, “linear perspective is a machine for annihilating reality” (2006: 93; cf. Koenderink et al., 2015; Rauschenbach, 1985). Or to soften the tone: the use of linear perspective annihilates some realities, omitting the possibility of their representation, while making three-dimensional aspects more salient. In other words, the use of a vanishing point hides a host of other things that could be represented, but now can’t be, once the demand for depth is introduced. However, despite this, representations that properly depict three dimensions are often seen as more veridical and true to reality, even though they also hide much. Naïve or “flat” representations—for example Egyptian or Byzantine art—are seen as distorting reality by omitting perspective altogether (Gombrich, 1956; Panofsky, 1991). The representation itself of course is not the reality, but merely a map of it (that is, it focuses us on some portions of reality and makes them salient).^14^

Consider how painting and fine art changed in the late 19th century when photography became available. Neurophysiologist Eric Kandel discusses how the work of artists at this time in Vienna “sought newer truths that could not be captured by the camera…[and] turned the artist’s view inward—away from the three-dimensional outside world and toward the multidimensional inner self” (Kandel, 2012: 4; also see Kandel, 2013). The camera could capture outward surfaces or “skins,” but not inward aspects that of course prove equally real. Artists such as Gustav Klimt “abandoned three-dimensional reality for a modern version of two-dimensional representation that characterizes Byzantine art” (Kandel, 2012: 113). Klimt captured the subject in flat, icon-like fashion, featuring symbolism and ornamentation. One form of representing reality (more photograph-like) is abandoned to give way to highlighting other aspects. The modernist mantra of the turn of the century Vienna—which united psychologists, artists, and neuroscientists alike—was that “only by going below surface appearances can we find reality” (Kandel, 2012: 16).^15^ Kandel suggests that it was this tradition, which “questioned what constitutes reality,” and which provocatively concluded that “there is no single reality,” that in fact gave rise to cognitive science and neuroscience (2012: 14, 113).^16^

The critical point here is that any visual scene can be represented in a number of different ways. We could compare different depictions of the same visual scene by, say, a photographer versus a photorealist, impressionist, surrealist, cubist, or symbolist painter. There is no sense in which one or another of these representations is more true to actual reality (cf. Koenderink et al., 2015). Each representation points to or expresses different aspects. Some aspects of a visual scene are made more salient by one depiction, necessitating the abandonment of others aspects. Surface appearances or three-dimensional realities might be foregone to capture other aspects. Even photographs are scarcely objective or neutral, as photos of the same visual scene can vary significantly—and thus capture different aspects of reality, hiding others—based on choices about aperture, shutter speed, and exposure (Koenderink, 2001). Any number of other technologies could be used to enhance, express, measure, elicit, or point out different features within a visual field.

At the most basic level, a painting can simply be described by what is physically there (Koenderink et al., 2014). Thus, prior to any demands for accurate depiction, we might objectively see a finished painting as constituted by its physical parts: a wooden frame, a canvas of some size, and the color pigment on the canvas.^17^ This is one description. The painting can also be considered more closely: the composition and arrangement of the pigments can be noted and perhaps some kind of judgment can be made about whether these appropriately capture, say, Euclidean space or perspective. This is another description, but not the only alternative. The list of possible demands for representation is too large to be captured on a two-dimensional surface. Of course, the most obvious problem in anchoring on the physical aspects of representation or perception is that it misses a wide swath of activity concerned with meaning and symbols. A painting is more than the sum of its physical elements, a canvas, and pigment. The way that the pigments are arranged, the subject matter of the representation, feature elements of meaning that scarcely can be captured in any physical way (Langer, 1953; Panofsky, 1955; 1991; also see Gormley, 2007). The arts teach us that a representational approach to perception cannot address how the physical things on a canvas—composed and arranged—elicit more than the luminance and other physical factors that could be measured. Recent work on Gestalt psychology reinforces this point (Wagemans et al., 2012).

Central to perception, then, is the “beholder’s share” (Gombrich, 1956). Observation is always theory-laden (Popper 1972) and there is no innocent eye that somehow directly captures or speaks truth to data or reality. The beholder’s share is not only captured by the species-specific nature of perception, but also by the experiences, theories, and insights that the beholder brings to any encounter. We might again cite Florensky, who argues that “the visual image is not presented to the consciousness as something simple, without work and effort, but is *constructed…*such that each of [image] is perceived more or less from its own point of view” (2006: 270; see also Panofsky, 1991).

Our argument is not merely a stylistic or artistic one, but it is directly applicable to science. What the arts reveal is that reality and perception is multifarious. We might, and perhaps should, observe and measure this multifariousness scientifically as well (Kandel, 2013; Koenderink, 2014). Many factors are not perceptible by the human eye, but nonetheless there. Science goes beyond naïve perception. We use all manner of perception-enhancing scientific tools and measurements to learn about the nature of reality. In all, the above research raises fundamental questions about the emphasis that Kahneman places on the “actual,” “physical,” and “veridical” aspects of reality (2003: 1453–1460). As we have discussed, perception simply does not give us this type of direct access to reality (cf. Frith, 2007), or certainly not to the type of singular, objective reality that Kahneman has in mind.

Furthermore, scholars interpret the fact that perception can be “primed” (for example by size, contrast, order), and that individuals can be led to see things in very different and discrepant ways, as evidence for bias (Kahneman & Frederick, 2002). The evidence from top-down priming is *not* evidence for bias, but rather evidence for the openness of reality to be interpreted and expressed in many different ways. What the arts illustrate is that rather than demand that subjects meet the requirements of, for example, linear perspective, there are a multitude of other demands that might also be made for representing, expressing, or seeing reality. Any single demand for verity is necessarily incomplete and illusory.

## Perception and rationality: So what?

Our arguments about perception may seem abstract and perhaps far removed from practical concerns about the study of rationality, of human judgment and decision-making. However, our thesis has significant implications.

First, there are two fundamentally different conceptions of human nature and rationality. One conception assumes that errors and mistakes are the critical phenomena to be demonstrated and explained (cf. Krueger & Funder, 2004). This literature uses the norm of omniscience as a convenient “null hypothesis”^18^—granting scientists themselves an all-seeing position—against which human decision making is measured. The conventional and even ritualistic use of this null hypothesis has endowed it a normative force. Yet, repeated rejections of this null hypothesis are of limited interest or concern when the normative status of the theory is itself questionable. We can only expect the list of deviations, biases, and errors to grow, indefinitely, without fresh theoretical light being shed. Unfortunately, many of these tests “reveal little more than the difficulty of the presented task” (Krueger & Funder, 2004: 322). The other approach to rationality focuses not on mistakes and error (from some omniscient norm), but on the nature of rationality itself. Such a theory needs to capture the accuracy manifest in human judgment (Jussim, 2015), as well as the fact that many of the seeming biases have heuristic value and lead to better judgments and outcomes (e.g., Gigerenzer & Brighton, 2009). Furthermore, this alternative theory needs to recognize that many of the simplistic tests of rationality omit important contextual information and also do not recognize that even simple stimuli, cues, and primes can be interpreted in many different ways. Thus, while psychology and behavioral economics can take credit for introducing psychological factors into judgment and decision making (cf. Thaler, 2015), we think that the literature cited here calls for a significant shift in the psychological assumptions about human nature.

We see both perception and rationality as a function of organisms’ and agents’ active engagement with their environments, through the probing, expectations, questions, conjectures and theories that humans impose on the world (Koenderink, 2012). The shift here is radical: from an empiricism that focuses on the senses to a form of rationalism that focuses on the nature, capacities, and intentions of the organisms or actors involved. While empiricism emphasizes the actual, physical characteristics within a visual scene (Kahneman, 2003), rationalism focuses us on the perceivers themselves. From this perspective, much of the work on bias, blindness, or bounded rationality—as we will illustrate next—can be interpreted quite differently. Research by developmental psychologists shows how even infants have *ex ante* theories or “core knowledge” about the world, which guide expectations and object perceptions (e.g., Spelke et al., 1992; also see Gopnik & Meltzoff, 1997), thus challenging empiricism and the overly strong focus on the senses.

We submit that a new generation of theories should start with a different premise, which grants human actors the same theoretical and scientific tools that we as scientists use to understand the world. The present asymmetry—between our assumptions about subjects versus the implied assumptions that we have about science itself—deserves attention. It has been touched on in economics, where Vernon Smith argues that “our bounded rationality as economic theorists is far more constraining on economic science, than the bounded rationality of privately informed agents” (2003: 526). When we experimentally whittle rationality down to the simplest of stimuli or cues, we lose valuable contextual information, held by these “privately informed agents,” which shapes perception and interpretation. The problem is that even the simplest of cues or stimuli afford wildly different interpretations.^19^ Thus the beliefs, ideas, conjectures and theories of agents deserve more careful attention. It is worth noting that this form of theorizing is scarcely new. It may be found in developmental psychology (e.g., Spelke et al., 1992) and in the history of philosophy, for example in the work of Plato, Kant, or Goethe. In the context of social science this premise links up with the type of theoretical endeavor envisioned by Adam Smith who argued that ultimately our theory of human nature and rationality—as paraphrased by Emma Rothschild—“must be a theory of people with theories” (2001: 50).

Second, our arguments might yield alternative interpretations to existing theories and experimental findings of bias, boundedness, or blindness. Part of our concern is that the findings of bias and error are affected by scientists’ own theoretical assumptions and expectations (cf. Bell, 1990),^20^ much like perceiving and awareness depend on people’s beliefs and expectations. If our theories postulate irrationality, and if we craft experimental tasks to prove this, we will find evidence for it. There is a large variety of stimuli that could be pointed to (and proven) but missed by human subjects in the lab or in the wild. But these types of findings can be interpreted in a number of different ways.

Consider a telling example. In their famous experiment on inattentional blindness, Simons and Chabris (1999) show how subjects miss a chest-thumping person in a gorilla suit walking across the scene, because these subjects were asked (primed) to count the number of basketball passes (cf. Chugh & Bazerman, 2007). Kahneman argues that the gorilla study points out something very fundamental about the mind, namely, that it is “blind to the obvious” (2011: 23–24). However, obviousness, from the perspective of perception—and awareness in particular—is far more complicated. If subjects were primed to look for the gorilla, and then asked to report on the number of basketball passes they observed, presumably they would also *not* be able to get the correct answer. Primes are equivalent to questions which direct awareness (cf. Koenderink, 2012), in the presence of visual fields that feature an extremely large (if not near infinite) variety of possible things that could be attended to. In the gorilla experiment, subjects could be asked to report on any number of things: the hair color of the participants, the gender or ethnic composition of the group, the expressions or emotion of the participants, the color of the floor, or whether they noticed what large letters were spray-painted on the wall (two large “S” letters). Any of these visual stimuli are evident—even obvious; *though only if you are looking for them (or not looking for something else)*. Missing any one of them is not blindness or bias—though the stimuli are evident and obvious—though it can be framed as such. Missing the gorilla is a success, given the task at hand. Thus these types of experiments provide evidence for the directedness of perception and awareness, and highlight how a very large set of things can be attended to and reported in any visual scene. Primes and cues (rightly) direct the attention and awareness of subjects.

In short, awareness and perception has little to do with the nature of the stimulus (Koenderink, 2012), even though this is the explicit assumption of behavioral work (Kahneman, 2003). What we are arguing for is thus a fundamentally different view of cognition. Awareness and perception are instead a function of the perceiver, of the questions, probes, and theories that any of us impose on even the simplest of visual scenes or surroundings, or on reality more generally. Shifting the emphasis to perceivers, rather than the nature of stimuli, provides a significant opportunity for future work.

Rationality and perception research has engaged in an exercise where scholars pre-identify and focus on a single percept or stimulus and then look for a common response, or point to a systematic deviation from a single, sought-after, rational answer (Koenderink, 2001). Of course, it is important that theories allow and dictate certain observations. But an *a priori* focus on irrationality leads to an unknown quantity of pre-publication trial-and-error of different experimental tasks, to find and report those results that indeed provide evidence of bias or illusion. Any number of tests and experiments could be devised to highlight irrationality, blindness, and bias—as even the simplest of visual scenes exhausts our abilities to describe it. Missing something obvious (and thus surprising) in a visual scene of course provides an important basis for publication. This tendency has been noted in the context of social psychology: “when judgments consistent with the norm of rationality are considered uninformative, only irrationality is newsworthy” (Krueger & Funder, 2004: 318). But again, the vast amount of decision making that humans get right receives little attention (e.g., Funder, 2012; Jussim, 2015). And, more importantly, the actual mechanisms of rationality and awareness never get addressed—a significant opportunity for future work.

The third, and perhaps most basic, implication of our arguments is that the rationality literature needs to rethink the multitude of visual examples and perceptual metaphors that are utilized to highlight bias. As we have discussed, visual illusions do not provide evidence of bias (Rogers, 2014; cf. Hoffman & Richards, 1984). Instead they reveal how the perceptual system works (well) in the presence of incomplete, degraded, or ambiguous input information (Koenderink, 2012; Zavagno et al., 2015). Visual illusions reveal that multiple responses, or ways of seeing, are equally rational and plausible, as highlighted in our discussion of the Ponzo illusion (see Fig. 1). Rational judgment, then, much like visual perception, can be seen as “multistable” (Attneave, 1971). As noted by Schwartz et al., “multistability occurs when a *single physical* stimulus produces alternations between different subjective percepts” (2012: 896, *emphasis added*). Whereas Kahneman and others working in the heuristics-and-biases tradition emphasize the “physical” or “actual properties of the object of judgment” (2003: 1453) and thereby focus on a single, fixed, and veridical interpretation (i.e., the rational response), we argue that even simple stimuli are characterized by indeterminacy and ambiguity. Perception is multistable, as almost any percept or physical stimulus—even something as simple as color or luminance (Koenderink, 2010)—is prone to carry some irreducible ambiguity and is susceptible to multiple different interpretations. Conscious perception is the result of ambiguity-solving processes, which themselves are not determined by the stimulus input. Similarly, the human susceptibility to priming and sensitivity to salient cues is not *prima facie* evidence of irrationality, but rather provides evidence of this multistability.^21^ Whether we are dealing with perception or reasoning, in information-deprived and ambiguous situations humans use whatever evidence or cues (or demand characteristics) are available to make judgments. This is also the basis for saying that apparent biases might be seen as rational and adaptive heuristics (Gigerenzer & Gaissmaier, 2011; McKenzie, 2003).

The specific opportunity for future research, suggested by our arguments, is to recognize the multistability and indeterminacy of judgment and rationality. Modal, average, or common responses can be useful for some purposes, but scholars might also take advantage of the large variance in judgments and use this information to understand heterogeneity in both perception and reasoning. The rationality literature has a tendency to label certain outcomes as biases or mistakes—and the catalogue of different biases now numbers in the hundreds. But this labeling has not allowed us to understand the actual *reasons* why humans behave in particular ways (Boudon, 2003). Furthermore, judgment and decision making often happen in ambiguous and highly uncertain environments, where specifying a single form of optimality is scarcely possible, though perhaps only with the benefit of hindsight. While the biases and bounded rationality literature is getting much traction in business and managerial literatures and settings, we wonder whether it even meaningfully applies to settings characterized by high levels of uncertainty (cf. Felin, Kauffman, Koppl, & Longo, 2014). It is precisely in these settings where the literature on rationality might in fact study how agent beliefs, expectations, and theories guide judgment and behavior, and how humans adjust as they make errors and learn from their behavior. Furthermore, the biases and rationality literatures have been extremely individualistic, scarcely accounting for the social dimensions of rationality. That is, human interaction in social, institutional, and organizational settings is likely to significantly shape how rationality “aggregates.” This is certain to be far more complicated than simple, linear addition, given complex, emergent outcomes. Thus, further theoretical and empirical attention is needed on the disparate social and organizational contexts within which judgment and decision making happen.

## Conclusion

The purpose of this paper has been to show how the bounded rationality and biases literature—in behavioral economics and cognitive psychology—has implicitly built its foundations on some problematic assumptions about perception. Arguments about perception are inadvertently interwoven into the rationality literature through the use of visual illusions, metaphors, and tasks, as examples of bias, boundedness, and blindness. The behavioral literature features an all-seeing view of perception, which we argue is untenable and in fact closely mirrors the assumption of omniscience which this literature has sought to challenge. We provide evidence from vision and perception science, as well as the arts, to make our point—along with suggesting some ways forward.

We hope that our arguments can help build a foundation for alternative ways of thinking about judgment, decision making, and rationality. Just as the perception literature—some of which we have cited—features a more pragmatic and multi-dimensional approach to seeing and vision, the rationality literature might also consider the “usefulness” (and striking successes) of human reasoning and judgment in disparate contexts that feature much ambiguity and possibility. The literature on “biases as heuristics” (Gigerenzer & Todd, 1999) has begun to move us in this direction, although it has also inherited some problematic assumptions about perception. But there is also an opportunity to study the varied organism-specific and contextual factors that shape human cognition and decisions in natural settings. Furthermore, human agents also actively engage with the world on the basis of their expectations, conjectures, and theories, which also provides a promising opportunity for future work. Most real-world settings feature a wild assortment of possible stimuli and cues, allowing for varied types of rationalities and interpretations (even of the same stimulus), thus requiring us to expand the scope of how rationality is specified, studied, and understood. If our suggested reorientation of the study of rationality takes hold, then it will move the literature toward recognizing cognition, judgment, and rationality as a multi-stable affair. We hope that our paper, while provocative, has at least opened up a conversation about the perception-rationality link and perhaps even a conversation about the very nature of rationality.

## Footnotes

1 As discussed by Vernon Smith (2003), perception was also central to Friedrich Hayek’s approach to rationality and economic theory.

2 Kenneth Arrow (1986) discusses how this assumption of rationality and individual homogeneity manifests itself in different ways in economics.

3 For a recent summary of the history of behavioral economics, see Thaler, 2015, 2016.

4 Models of search indeed are ubiquitous across the cognitive and social sciences (for an overview, see Hills et al., 2015; also see Abbott et al., 2015; Hills et al., 2012).

5 This idea of an ideal observer can be reasonable in highly restricted settings of psychophysics. Low-level vision is limited by photon statistics, and visual acuity by the wavelength of electromagnetic radiation and photon statistics. Here vision is indeed limited by the physics and the ideal observer is easily defined and useful. We can also do so in acoustics. But there is no way to conceive of ideal observers where meaning and awareness is concerned, the “available information” is actually “structural complexity” in the Shannon sense. However, these models are about vision as a physiological device, again, ignoring awareness.

6 Simon briefly mentions that “we are not interested in describing some physically objective world in its totality, but only those aspects of the totality that have relevance as the ‘life space’ of the organisms considered” (1956: 130). However, there is no subsequent discussion of organism-specific factors related to this life space of organisms, neither in his early or his later work. The emphasis is on universal factors that apply across species (see Simon, 1990).

7 Simon remarked to a friend that we need a “less God-like and more rat-like chooser” (Crowther-Heyk, 2005: 6). These types of arguments link with behaviorism, which emphasized environments over organisms: “the variables of which human behavior is a function lie in the environment” (1977: 1). Skinner further argued that the "the skin is not that important as a boundary" (1964: 84). Behaviorism also focused heavily on environments and external stimuli, at the expense of understanding the (comparative) nature of the organism.

8 Bayesian models of cognition and rationality have of course been criticized in the literature, for their seeming similarities to behaviorism, lack of attention to underlying mechanisms, their equating rationality with computation, lack of empirical findings, and overly strong focus on rationality, etc. (e.g., Bowers & Davis, 2012; Jones & Love, 2011). Our focus here is different, in that we highlight the perception and vision-related assumptions made by this literature.

9 This intuition has been used to highlight incoherent judgments in law (Sunstein, Kahneman, Schkade, & Ritov, 2002).

10 Goethe famously captured this intuition: “a grey object on a black ground appears much brighter than the same object on a white ground. If both comparisons are seen together the spectator can hardly persuade himself that the two greys are identical” (1840: 15 – see Wade, 2014: 860).

11 What is interesting is that context-dependence is emphasized in the luminance task, but that same context-dependence is not, as illustrated by the Ponzo illusion, recognized in the perspective-based task. The evidence of bias is used selectively.

12 For further discussion and debate about the interface theory of perception, see a recent set of articles published in *Psychonomic Bulletin & Review* (Hickok, 2015).

13 We can reduce anything into “pointer readings” (Eddington, 1927; 247–252; also Koenderink 2012)—measurements of size, position or motion—though the actual nature of objects (and particularly how we perceive these objects) is far more complex, and further it also depends on perspective and the observer. And, beyond any pointer readings, complexity is emergent beyond any physical factors that might be measured (Ellis, 2005).

14 Similarly, in the context of geography, maps can use a linear, geometric approach to representing the world, though alternative approaches are also readily available (e.g., Dora, 2013).

15 This has also been discussed in sculpture. For example, Antony Gormley highlights the need to move beyond outward appearances to express the “other side of appearance, from inside the skin” (2007: 1515; cf. Hildebrand, 1907).

16 Eric Kandel highlights how this modernist ethos—the inward turn from mere surface appearances to deeper realities—“would stretch from medical clinics and consulting rooms to artists’ studios and finally to neuroscience laboratories” (2012: 16).

17 As vividly remarked by Maurice Denis (1890: 540), “remember that a picture, before being a battle horse, a nude, an anecdote or whatnot, is essentially a flat surface covered with colors assembled in a certain order.”

18 In his recent book on behavioral psychology and economics, Richard Thaler recounts what psychologist Thomas Gilovich said to him: “I never cease to be amazed by the number of convenient null hypotheses economic theory has given you” (Thaler, 2015: 97). The problem is that omniscience indeed is an all too convenient null hypothesis, which is easy to demonstrate as false, in an infinite variety of ways. However, beyond continuing to point to deviations from this convenient null hypothesis, future work also needs to more proactively account for what rationality is.

19 Koenderink (2012: 175) captures this as follows: “The same sequence of keyboard presses may be interpreted as a password, a number, a word in the English language, some code, an assembler command, gibberish. . . Input structure is not intrinsically meaningful, meaning needs to be imposed (magically) by some arbitrary format.” Thus formats (in the case of computer science: Knuth, 1997) can be seen as theories about how to make sense of and interpret a set of (even similar) inputs.

20 As Einstein put it, “whether you can observe a thing or not depends on the theory which you use. It is the theory which decides what can be observed” (quoted in Polanyi, 1971: 604; cf. Popper 1979).

21 Of course, not just anything is “prime”-able or susceptible to so-called “top down” (e.g., categories or language) effects on perception (as discussed by Firestone & Scholl, 2015). However, our emphasis is on the fact that most perceptual cues and stimuli can be interpreted in different ways, far from yielding singular responses. The use of attentional cues or primes in experiments merely is a (adaptive and rational) response to having to deal with ambiguous stimuli in uncertain environments.
